# Disseminated bronchiectasis in an adult with common variable immunodeficiency

**Published:** 2015-03-30

**Authors:** Andrés Felipe Zea-Vera, Olga Lucia Agudelo-Rojas

**Affiliations:** 1 Department of Internal Medicine, Faculty of Health, Universidad del Valle. Cali, Colombia; 2 Research Group VIREM, School of Basic Sciences, Faculty of Health, Universidad del Valle. Cali, Colombia

**Keywords:** Primary immunodeficiency, hypogammaglobulinemia, common variable immunodeficiency, bronchiectasis, recurring pneumonia

## Abstract

Primary immunodeficiencies (PID) are traditionally considered childhood diseases; however, adults account for 35% of all patients with PID. Antibody deficiencies, especially Common Variable Immunodeficiency (CVID), which have their peak incidence in adulthood, require a high suspicion index. Even though the estimated frequency of CVID is not high (1:25,000), high rates of under diagnosis and under reporting are very likely. The delay in diagnosis increases the morbidity and mortality; therefore, adult physicians should be able to suspect, identify and initiate management of individuals with PID. Here we report the case of a 37 year-old man presenting to the emergency room with dyspnea, fever and cough; he developed respiratory failure requiring mechanical ventilation. He complained of recurring pneumonia associated with widespread bronchiectasis since he was 18 years old. Serum immunoglobulins quantification showed severe hypogammaglobulinemia (total IgG <140 mg/dL; total IgA, 2.9 mg/dL; and total IgM <5 mg/dL). Treatment with Human Intravenous Immunoglobulin (IVIG) 10% was started, and with antibiotic treatment for severe pneumonia (during 14 days) was also prescribed. His clinical evolution has been favorable after one year follow-up. Common Variable Immunodeficiency (CVID) diagnosis was made.

## Abstract (es)

Las inmunodeficiencias primarias (IDP) son patologías que tradicionalmente se consideran de la niñez sin embargo los adultos representan el 35% del total de pacientes con IDP. Las deficiencias de anticuerpos, en especial la Inmunodeficiencia Común Variable (IDCV) tienen su pico de incidencia en la edad adulta, requiere un alto índice de sospecha y si bien su frecuencia estimada no es alta (1:25,000), es muy posible que el subregistro y subdiagnóstico si lo sean. El retraso en el diagnóstico aumenta la morbi-mortalidad razón por la cual los médicos de adultos deben estar en capacidad de sospechar, identificar e iniciar el manejo de las personas con IPD. Presentamos el caso de un hombre de 37 años de edad atendido en la sala de urgencias con disnea, fiebre y tos, desarrolla falla respiratoria requiriendo ventilación mecánica. Refería neumonías a repetición desde los 18 años de edad asociadas con bronquiectasias generalizadas. La cuantificación de inmunoglobulinas séricas evidenció hipogammaglobulinemia severa (IgG total <140 mg/dL, IgA total 2.9 mg/dL, IgM total <5 mg/dL), se inició inmunoglobulina humana endovenosa (IGIV) al 10%, y recibió tratamiento antibiótico por 14 días para neumonía severa, su evolución clínica ha sido favorable hasta ahora (un año de seguimiento), se estableció el diagnostico de Inmunodeficiencia Común Variable (IDCV).

## Introduction

Common Variable Immunodeficiency (CVID) is a predominantly antibody primary immunodeficiency in which the humoral immune response is altered 1,2. The clinical spectrum of this disease ranges from repeated infections with sequelae such as the appearance of bronchiectasis, to the development of malignancies or autoimmunity. Despite being a genetic disorder, adults are the most affected, so efforts should be attempted to educate medical community 2,3. Here we present the case of a 37-year-old man with recurrent sinopulmonary infections and widespread bronchiectasis, in whom a severe hypogammaglobulinemia with symptoms compatible with Common Variable Immunodeficiency was demonstrated.

## Case description

A 37-year-old man presented to the emergency department of a level III hospital in the city of Cali (Colombia) complaining of respiratory distress, fever and cough with greenish expectoration of approximately one week duration, with worsening dyspnea in the past 48 hours until being unable of performing any minimal effort. At admission, he presented hypotension (78/36), tachycardia (126 beats/min), and tachypnea (38 breaths/min), with saturation of 76% O_2_ (O_2_ atmosphere); lung auscultation revealed multiple over-aggregate and overall decreased breath sounds. The patient reported having immunodeficiency antibody. Few minutes after admission, he presented respiratory failure requiring intubation and vasoactive support with mechanical ventilation. On suspicion of septic shock, antibiotic coverage was initiated with vancomycin and cefepime, after taking blood cultures.

**Figure 1.**
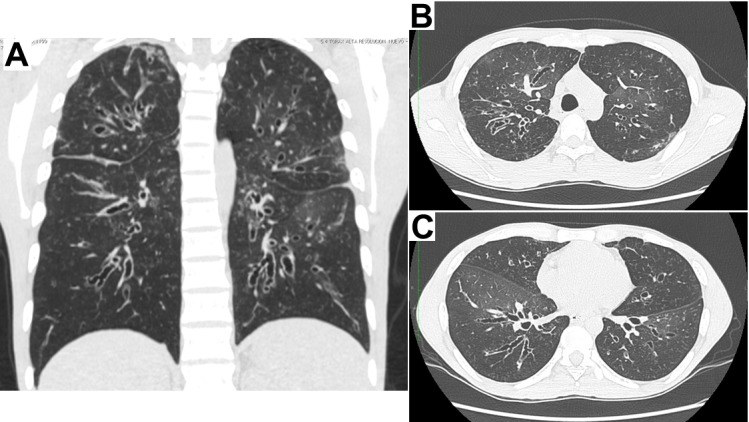
High resolution chest scans which show widespread bronchiectasis in the four quadrants (A); and in the cross sections at the level of the aortic arch (B), and the left ventricle (C).

The patient is native to, and came from Cali (Valle province, in Colombia). As relevant background, he refers pneumonia, sinusitis and recurrent otitis since he was aged 18 yrs, with countless episodes (6 to 10 per year) requiring long courses of oral or intravenous antibiotics and multiple hospitalizations. Since 2002 cylindrical and cystic bronchiectasis had been documented in all four quadrants (Fig. 1A), equally documented in the cross sections at the level of the aortic arch and the left ventricle (Fig. 1B y1C).

Studies for cystic fibrosis and autoimmunity were performed with negative results. In 2006, he received six months of supervised shortened treatment for pulmonary tuberculosis, diagnosed by positive sputum culture at 12 weeks (serial smear negative), with microbiological cure. He had consulted countless times different specialties and subspecialties of internal medicine, and despite having documented very low titers of total serum immunoglobulins (at least 5 yrs prior to this hospitalization), for unclear reasons he did not receive any replacement therapy. Tests for HIV antibodies were performed for over 7 times with negative results.

Paraclinical income tests showed leukocytosis (17,820/mm^3)^ at the expense of neutrophilia (79%), major bandemia (11%) without anemia (Hb 13.8 g/dL), normal platelets (281,000/mm^3)^, and elevated acute phase reactants (94 CRP mg/L). Kidney function was normal, with no proteinuria; however, the patient had a markedly hypoproteinemia at the expense of globulins (total protein 4.8 g/dL albumin 3.6 g/dL, globulins 1.2 g/dL). Quantification of total serum immunoglobulins by nephelometry showed severe hypogammaglobulinemia (Total IgG <140 mg/dL, Total IgA, 2.9 mg/dL; Total IgM <5 mg/dL) with an electrophoresis protein value compatible with agammaglobulinemia. The same day of admission, intravenous human immunoglobulin (IVIG) was initiated at a rate of 800 mg/kg. Mechanical ventilation was required for 5 days, with no pathogen documented in the cultures. The patient received outpatient oral antibiotics, for which he completed 14 days of empirical antibiotic therapy. 

**Figure 2.**
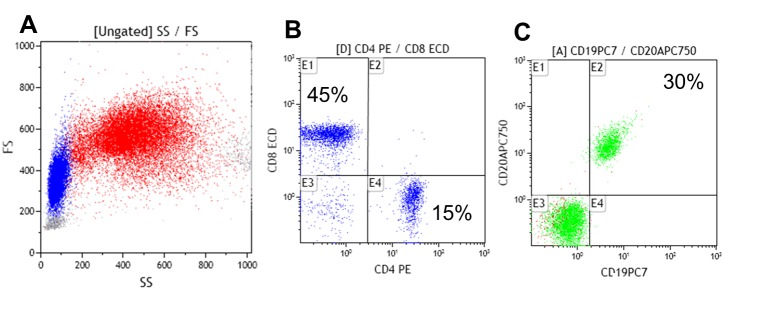
Quantification of T lymphocytes (CD4^+^/CD8^+^ and B lymphocytes (CD19^+^/CD20^+)^ populations by flow cytometry in peripheral blood. Analysis of dot plot size and complexity (Forward and Side scattered) of total leukocytes (A), quantification of CD4^+^ T lymphocytes and CD8^+^ T lymphocytes (B), and total B lymphocytes quantification (C). Note the reversal of the CD4/CD8 ratio, 1:3 (instead of 2:1) and the percentage increase of 30% (6-19%) in B Lymphocytes.

The patient was examined by the Immunology service prior to the start of IGIV, it was found that both the responses to vaccine antigens and infectious pathogens were absent; anti-core antibodies (Anti core-HBV) and antibodies to Hepatitis B surface antigen were determined (Anti-HBsAg), as well as fourth generation EIA for HIV, total antibodies against hepatitis C virus (HCV), IgM and IgG antibodies against cytomegalovirus (CMV), herpes simplex IgG for 1-2 virus (HSV), and IgG antibodies against tetanus and rubella. The findings demonstrated not only the (specific) quantitative deficiency but also a defective functional humoral immune response. Evaluation of lymphocyte subpopulations in peripheral blood showed an absolute B lymphocyte count of 420 normal cells/µL (100-500) (Fig. 2C), accompanied by inversion of the lymphocyte T CD4^+^/CD8^+^ ratio, at the expense of a decrease in CD4^+^ T lymphocytes to 190 cells /L (300-1,400) (Fig. 2B).

At present, the patient receives IGIV 10% at a dose of 800 mg/kg every 4 weeks, he receives prophylaxis with TMP/SX 160/800 mg every other day, he completed a cycle of 22 sessions of pulmonary rehabilitation, and he is managed jointly by the Immunology, Pneumology and Internal Medicine services. His evolution has been satisfactory during the last year after the start of replacement therapy with IVIG, the patient had only one episode of pneumonia that required a short course of levofloxacin with excellent response; however, his lung capacity is very limited, which requires continuous oxygen therapy. The clinical picture of this patient, the serologic findings, the low titers of serum immunoglobulins, and the presence of B lymphocytes (CD19^+^/CD20^+)^ in peripheral blood determine a diagnosis of Common Variable Immunodeficiency (CVID).

## Discussion

Primary immunodeficiencies (PID) are considered rare diseases (prevalence 1: 25,000 to 1: 50,000), which results in a significant delay in diagnosis that leads to impoverished prognosis, increased comorbidities and clinical worsening, and increased cost to the health system for not being this condition timely identified 4. Traditionally, it has been accepted that PID are diseases of childhood, so internal medicine rarely addresses them, which contributes to the fact that adult doctors are not familiar with this disorder 5.

Common Variable Immunodeficiency (CVID) is the primary immunodeficiency of clinical relevance most frequently found in adulthood 6,7. Traditionally, it is accepted that this disease has two peaks of incidence, the former in childhood and the latter between the second and third decades of life, as it is the case of our patient. The delay in diagnosis is common, occurring 2, 15 or even 20 yrs after the onset of symptoms 7.

Common Variable Immunodeficiency is a very heterogeneous disease in which numerous mutations associated with maturation or activation of B lymphocytes have been identified, which ultimately results in both a quantitative and qualitative inability to produce immunoglobulin genes. Today, two main clinical spectra are recognized: uncomplicated CVID, referred to the "classical" patient with repetitive sinopulmonary infections or gastrointestinal complications; and complicated CVID, in which recurrent infections occur, accompanied by visceromegalies (hepato/splenomegaly), lymphadenopathy, autoimmunity (usually cytopenias), and lymphomas 3. The presence of widespread bronchiectasis, as in the case of our patient, requires an active seeking and ruling out of primary immunodeficiencies, as it has been shown that up to 10% of patients with bronchiectasis (not caused by cystic fibrosis) correspond to antibody deficiencies, especially CVID 8,9.

The diagnostic criteria for CVID were established by the European and Panamerican Immunodeficiencies Societies (ESID/PAGID) in 1999, and still in force so far, include: marked decrease (at least 2 standard deviations below the mean for age) of IgG, IgA and/or IgM in serum; to be older than 4 yrs, to be negative for isohemagglutinins and/or to have poor responses to vaccines; besides, other causes of hypogammaglobulinemia must have been excluded. Compliance with all of the above criteria is essential for diagnosis prior to initiation of therapy with IVIG replacement, because this therapy modifies serological parameters up to six months after the last application 10.

The presence of lymphopenia at the expense of T CD4^+^ lymphocytes can be an important confounding factor, since most of the treating physicians associate this finding with HIV/AIDS, as in this case. Importantly, up to 25% of patients with CVID present with low LT CD4^+^, without commitment of cellular immunity.

The goal of treatment is replacement of the humoral response by administering human immunoglobulin in order to reduce infectious and autoimmune complications, and the emergence of granulomas or malignancy 11. In Colombia different forms of human immunoglobulin are available, and they are authorized by INVIMA for use in patients with antibody deficiencies. The IVIG must be administered at a dose between 400-800 mg/kg every 3-4 weeks, depending on the valley levels and the clinical response; we also have subcutaneous human immunoglobulin (SubQ) for weekly application between 100-200 mg/kg dose. The use of antibiotic prophylaxis is not clearly established; however, it is recommended the use of macrolides and quinolones; and for cases that present with low CD4^+^ T lymphocytes counts, it is recommended prophylaxis with trimethoprim/sulfamethoxazole 12,13.

Primary immunodeficiencies in adults must be a diagnostic possibility, and in Colombia, the technologies and methodologies to confirm the diagnosis are available 14. This case report aims to draw attention to a disease that requires a high index of suspicion, and the low reported prevalence is possibly due to underreporting and under diagnosis, which has an impact on the prognosis of the disease.
